# Winter Butter

**Published:** 1856-03

**Authors:** 


WINTER BUTTER.—In many parts of our country the art of making good butter in winter is very imperfectly understood, and by some dairy-women thought to be entirely impossible. But it can be done in December as well as in May. The plan of doing it is this: The cows should be stabled and fed on sweet hay and other provender. Instead of keeping the milk in a warm place, it should be put in a cold one, and no matter how soon it freezes, because freezing it will separate the cream much more perfectly than it will rise without this atmospheric temperature, and it can then be taken off with less trouble. And when the cream is churned the churn should not be placed near a fire; the ordinary heat of a kitchen would be sufficient. Too much warmth destroys both the complexion and the flavor of butter. In the winter butter requires more time in churning than in summer; but when patience assists the laborer, the task is made no task at all.

Butter cured with half ounce of salt, quarter ounce of saltpetre, quarter ounce of moist sugar, pounded, used in the proportion of an ounce to each pound of butter, will be found to keep good a longer time, and have a more delicious flavor than when salted in the ordinary way.

